# The Clinical and Angiographic Outcomes of Postdilation after Percutaneous Coronary Intervention in Patients with Acute Coronary Syndrome: A Systematic Review and Meta-Analysis

**DOI:** 10.1155/2021/6699812

**Published:** 2021-04-09

**Authors:** Yan Li, Xiying Liang, Wenjiao Zhang, Xuan Qiao, Zhilu Wang

**Affiliations:** ^1^The First Clinical Medical College of Lanzhou University, Lanzhou, Gansu, China; ^2^Department of Cardiology, The First Hospital of Lanzhou University, Lanzhou, Gansu, China

## Abstract

**Objective:**

The effect of postdilation in patients with acute coronary syndrome is still controversial. This meta-analysis aims to analyze the clinical and angiographic outcomes of postdilation after percutaneous coronary intervention in patients with acute coronary syndrome.

**Methods:**

PubMed, Embase, the Cochrane Library, Web of Science, CNKI, and Wangfang databases were searched from inception to August 30, 2020. Eligible studies from acute coronary syndrome patients treated with postdilation were included. The primary clinical outcome was major adverse cardiovascular events (MACE), the secondary clinical outcomes comprised all-cause death, stent thrombosis, myocardial infarction, and target vessel revascularization, and the angiographic outcomes were no reflow and slow reflow.

**Results:**

11 studies met inclusion criteria. In clinical outcomes, our pooled analysis demonstrated that the postdilation had a tendency of decreasing MACE (OR = 0.67, 95% CI 0.45–1.00; *P* = 0.05) but significantly increased all-cause death (OR = 1.49, 95% CI 1.05–2.12; *P* = 0.03). No significant difference existed in stent thrombosis (OR = 0.71, 95% CI 0.40–1.26; *P* = 0.24), myocardial infarction (OR = 1.40, 95% CI 0.51–3.83; *P* = 0.51), and target vessel revascularization (OR = 0.61, 95% CI 0.21–1.80; *P* = 0.37) between postdilation and non-postdilation groups. In angiographic outcomes, there were no significant differences in no reflow (OR = 1.19, 95% CI 0.54–2.65; *P* = 0.66) and slow reflow (OR = 1.12, 95% CI 0.93–1.35; *P* = 0.24) between two groups.

**Conclusions:**

The postdilation tends to reduce the risk of MACE but significantly increases all-cause death, without significantly affecting stent thrombosis, myocardial infarction, target vessel revascularization, and coronary TIMI flow grade. However, more randomized controlled trials are required for investigating the effect of postdilation for patients with acute coronary syndrome (registered by PROSPERO, CRD42020160748).

## 1. Introduction

Percutaneous coronary intervention (PCI) has been widely used for patients with acute coronary syndrome (ACS), and the optimum coronary stent deployment is crucial to improve prognosis in the current practice of PCI. Stent underexpansion is usually the failure to achieve a minimal in-stent dimension of more than 80% of the average reference segment diameter in patients with PCI. Studies showed that late stent thrombosis and very late stent thrombosis are mainly related to malapposition (31%), while prominent mechanisms of acute stent thrombosis and subacute stent thrombosis are malapposition (48%) and underexpansion (26%) [1]. Underexpansion is a significant cause of restenosis [2]. Thus, the postdilation of stent deployment is performed to achieve optimal stent expansion and complete the apposition of stent struts against the vessel wall [3, 4]. Studies indicated that the postdilation with a noncompliant balloon at higher pressure could reduce the restenosis rate and improve minimal stent area and minimal lumen diameter in unselected patients with stents implantation [3, 5]. Prolonged inflation could increase stent expansion and strut apposition [6], although overexpansion could increase neointimal hyperplasia caused by the inflammatory response to vessel wall injury and lead to an increased incidence of periprocedural myocardial infarction due to thrombus or plaque debris embolization in patients except for myocardial infarction and restenosis of the coronary artery [7–9]. A recent meta-analysis demonstrated that the postdilation of stent deployment did not improve clinical outcomes in patients with coronary artery disease, which suggested that the strategy should be selectively employed after stent implantation [10]. However, compared with stable coronary artery disease, the patients with ACS may have a higher risk of in-stent thrombosis due to increased platelet reactivity, lack of endothelialization of vascular endothelium, delayed healing, and exposure to inflammation and coagulation environment [11]. In recent years, several studies suggested that the postdilation reduced target vessel revascularization [12, 13], while others suggested that the strategy increased death [14, 15]. The benefits of postdilation in patients with ACS remain controversial. Therefore, a hypothesis that the postdilation is feasible after stent implantation in patients with ACS was made. This meta-analysis was conducted to verify the hypothesis that the postdilation could improve clinical and angiographic outcomes in patients with those.

## 2. Methods

### 2.1. Search Strategy and Eligible Criteria

The systematic review and meta-analysis was performed in accordance with the reporting items for systematic review and meta-analysis guidelines [16]. The review protocol was registered by PROSPERO, CRD42020160748. A literature search was systematically performed in PubMed, Embase, The Cochrane Library, Web of Science, CNKI, and WANGFANG databases from inception to August 30, 2020, using the following terms “acute coronary syndrome” OR “ST-segment elevation myocardial infarction” AND “percutaneous coronary intervention” OR “angioplasty” AND “post-dilation” without restrictions on region, publication type, or language. Moreover, relevant reviews and meta-analyses to identify other eligible studies were searched manually. The following criteria had to be met to consider a study qualified for this meta-analysis: (1) all patients presenting with ACS including ST-segment elevation myocardial infarction (STEMI), non-ST-segment elevation myocardial infarction (NSTEMI), and unstable angina; (2) reporting coronary thrombolysis in myocardial infarction (TIMI) flow grade or one of the following clinical outcomes: MACE, all-cause death, stent thrombosis, myocardial infarction, and target vessel revascularization; (3) comparison of postdilation group and non-postdilation group; (4) randomized controlled trials or observational studies; (5) only studies enrolling patients with ACS included, studies involving other patients excluded.

### 2.2. Outcomes and Definitions

The primary clinical outcome was MACE. The secondary clinical outcomes were composed of all-cause death, stent thrombosis, myocardial infarction, and target vessel revascularization. The definition of composited outcome, MACE, was derived from original studies (Supplementary Table S1). All-cause death was defined as death caused by any reason including cardiac death and noncardiac death. The stent thrombosis was defined as definite, probable, or possible thrombosis [17]. The angiographic outcome was coronary TIMI flow grade after the postdilation. TIMI 0-1 flow was defined as no reflow and slow reflow was defied as failed to achieve TIMI 3 flow.

### 2.3. Data Extraction and Quality Assessment

Two researchers (LY and LXY) independently reviewed the titles, abstracts, and full-texts of all searched literature to determine eligible studies. In addition, the baseline characteristics, procedural characteristics, and outcomes were extracted by the two researchers separately. A standard data extraction form was designed before extraction. The risk of bias was appraised by the other two researchers (ZWJ and QX) independently in the method and result section. Any differences or uncertainties shall be resolved by consensus or, if necessary, by a third party (WZL). The Cochrane tool of collaboration was used for the quality assessment of randomized controlled trials [18] and the Newcastle–Ottawa scale [19] for observational studies. It should be resolved through negotiation when there were divergences and, if necessary, interfered by a third party (WZL). As all analyses were based on previously published studies, ethical approval and informed consent of patients are exempt.

### 2.4. Statistical Analysis

Review Manager Version (RevMan) 5.3 (The Nordic Cochrane Center, The Cochrane Collaboration, 2014, Copenhagen, Denmark) and Stata Version 12.0 (STATA Corporation, College Station, TX, USA) were used to perform statistical analysis. Continuous variables of baseline characteristics were presented as mean ± standard deviation (SD). Dichotomous variables were presented as count or percentages. All outcomes were calculated with odds ratio (OR) of DerSimonian and Laird and 95% confidence intervals (95% CI) by means of Mantel-Haenszel method. All tests were two-sided *P* values, and a *P* < 0.05 was considered statistically significant. Heterogeneity of the eligible studies was assessed by the Cochrane *Q* statistic with Pearson chi-square test and the Higgins *I*^2^ test. Random-effects model was performed to calculate the pooled OR if there was a significant heterogeneity (*I*^2^ ≥ 50%); otherwise fixed-effects model was used. Sensitivity analysis was carried out to evaluate its impact on pooled value by excluding each study when heterogeneity was obvious (*I*^2^ ≥ 50%) and subgroup analysis was performed to explain sources of heterogeneity. The outcome was analyzed by an intention-to-treat analysis. Publication bias test will not be performed in less than 10 studies.

## 3. Results

### 3.1. Search Results and Study Characteristics

**Figure 1:**
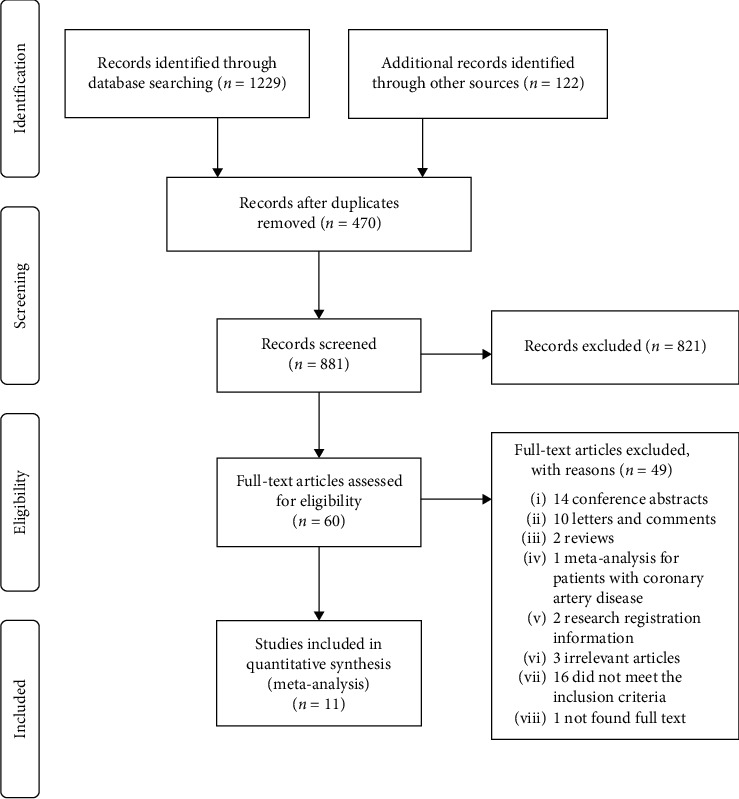
Flow chart of study selection.

The literature search yielded 1351 articles, 821 of them were excluded after screening titles and abstracts, and 60 of full-texts were reviewed. Ultimately, 11 studies (eight observational studies and three randomized controlled trials) enrolling 5663 patients met the inclusion criteria and are included in this meta-analysis (Figure 1) [12–15, 20–26]. All studies were published between 2010 and 2019; six of them were multicenter studies and five were single-center studies. Among them, seven studies provided data of clinical and angiographic outcomes, two studies provided only clinical outcomes, and two studies provided only angiographic data. Among all eligible patients, there were 4347 (76.8%) patients with STEMI and the rest of them were patients with ACS including STEMI, NSTEMI, and unstable angina. The postdilation strategy of stent deployment was received in 2514 (44.4%) of all patients, and 1937 (77.0%) of them were patients with STEMI. However, 3149 (55.6%) of all patients did not receive the postdilation strategy, of whom 2,410 (76.5%) were patients with STEMI. The sample sizes of studies varied from 124 to 1358. The majority of patients were males with age varying from 56.4 to 63.6 years. Hypertension accounted for 54.3% of all patients, diabetes mellitus 22.5%, smokers 30.6%, and dyslipidemia 47.2%. The overwhelming majority of the studies used drug-eluting stents; others used bare-metal stents and bioabsorbable scaffolds. The drug-eluting stents were used in four studies, the bioabsorbable scaffolds were used in one study, and the joint application of bioabsorbable scaffolds and drug-eluting stents was used in one study. The duration of follow-up ranged from one month to five years. The baseline and procedural characteristics of studies included are presented, respectively (Table 1 and Table 2). Quality assessments of the studies included are reported (Supplementary Figure S1 and Table S2).

### 3.2. The Primary Clinical Outcome

**Figure 2:**
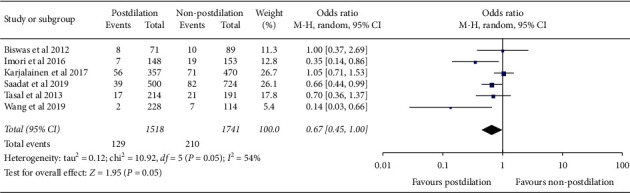
Forest plot of primary clinical outcome between postdilation and non-postdilation group. Notes: MACE = major adverse cardiac events.

The risk of MACE was reported in six studies and there was a decreasing trend after postdilation (OR = 0.67, 95% CI 0.45–1.00; *P* = 0.05, *I*^2^ = 54%) (Figure 2). The sensitivity analysis indicated that postdilation reduced the incidence of MACE after omitting one study (OR = 0.58, 95% CI 0.38–0.89; *P* = 0.01, *I*^2^ = 36%) [14] (Supplementary Figure S2(a) and Figure S3(a)). The subgroup analysis showed that there was no significant difference between the two groups after regrouping according to classification of diseases (STEMI or any ACS) and duration of follow-up (<12 months or ≥12 months) (Supplementary Figure S4(a)).

### 3.3. The Secondary Clinical Outcomes

**Figure 3:**
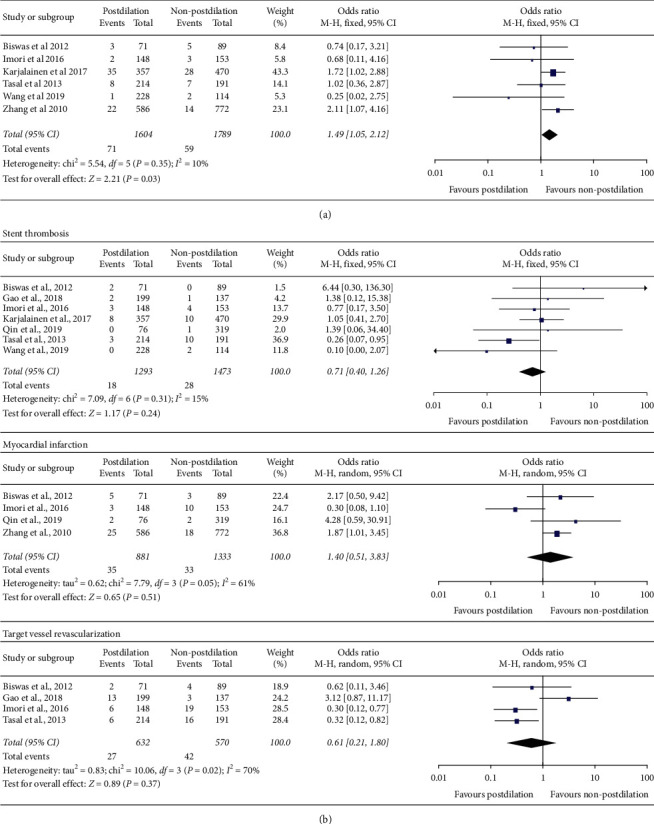
Forest plots of secondary clinical outcomes between postdilation and non-postdilation groups.

The risk of all-cause death is higher in postdilation group than that in non-postdilation group in patients with ACS (OR = 1.49, 95% CI 1.05–2.12; *P* = 0.03, *I*^2^ = 10%) (Figure 3(a)), but there are no significant differences in stent thrombosis (OR = 0.71, 95% CI 0.40–1.26; *P* = 0.24, *I*^2^ = 15%), myocardial infarction (OR = 1.40, 95% CI 0.51–3.83; *P* = 0.51, *I*^2^ = 61%), and target vessel revascularization (OR = 0.61, 95% CI 0.21–1.80; *P* = 0.37, *I*^2^ = 70%) (Figure 3(b)). There was obvious heterogeneity in myocardial infarction (*I*^2^ = 61%) and target vessel revascularization (*I*^2^ = 70%). Two studies producing heterogeneity were determined by sensitivity analysis [12, 23] (Supplementary Figure S2(b) and Figure S2(c)). The heterogeneity decreased and the statistical significance changed (OR = 2.03, 95% CI 1.18–3.50; *P* = 0.01, *I*^2^ = 0%) (Supplementary Figure S3(b)) after removing the study [12], which suggested that the postdilation increased the incidence of myocardial infarction. The heterogeneity and statistical significance of target vessel revascularization also changed after omitting the study of Gao et al. [23] (OR = 0.34, 95% CI 0.18–0.63; *P* = 0.0007, *I*^2^ = 0%) (Supplementary Figure S3(c)), indicating that the postdilation decreased the incidence of target vessel revascularization. There were no significant differences in stent thrombosis and myocardial infarction in two groups when subgroup analysis was carried out according to classification of diseases and duration of follow-up. The postdilation did not affect all-cause death in patients with STEMI but reduced the risk of target vessel revascularization in patients with any ACS (Supplementary Figure S4(b)). Furthermore, the postdilation did not increase the risk of all-cause death but reduced the risk of target vessel revascularization in patients with ACS within 12 months when regrouping according to duration of follow-up (Supplementary Figure S4(c)).

### 3.4. The Angiographic Outcomes

**Figure 4:**
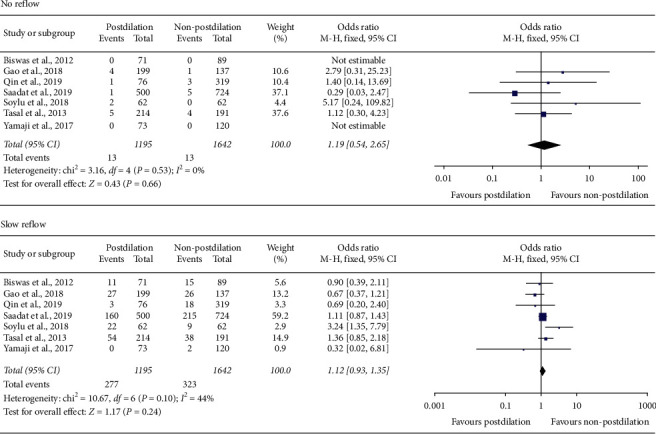
Forest plots of angiographic outcomes between postdilation and non-postdilation groups.

The no reflow and slow reflow were reported in seven studies involving 2837 patients with ACS, which indicates that there were no significant differences between postdilation and non-postdilation groups (OR = 1.19, 95% CI 0.54–2.65; *P* = 0.66, *I*^2^ = 0%; OR = 1.12, 95% CI 0.93–1.35; *P* = 0.24, *I*^2^ = 44%) (Figure 4).

## 4. Discussion

This systematic review and meta-analysis first assesses the clinical and angiographic outcomes of postdilation after coronary stent implantation in patients with ACS, which shows that the postdilation of stent deployment has a tendency of reducing the risk of MACE but significantly increases all-cause death and there is no significant difference in stent thrombosis, myocardial infarction, and target vessel revascularization of clinical outcomes. In addition, the rates of no reflow and slow reflow in postdilation group are similar to those in non-postdilation group.

The ESC guideline recommended that the majority of patients with ACS should use the invasive PCI, and primary PCI is the preferred reperfusion strategy for STEMI patients [27]. In the bare-metal stents era, the restenosis rate caused by neointimal hyperplasia was between 20% and 30% [28]. With the advancement of stents technology, the drug-eluting stents improve restenosis compared with bare-metal stents [29]. However, complications after stent implantation, such as in-stent thrombosis, no reflow, and others still occur. The majority of nonfatal myocardial infarction and 45% of death were included in the clinical sequelae of stent thrombosis [30]. The postdilation is a treatment strategy with noncompliant balloon of appropriate size [31], which could improve stent underexpansion and incomplete stent apposition, in turn reducing in-stent restenosis and target vessel revascularization [3]. The POSTIT trial, aiming to evaluate the necessity of postdilation after coronary stent deployment, manifested that only 29% of patients achieved the optimum stent deployment (minimal stent diameter ≥90% of the average reference lumen diameter assessed by intravascular ultrasound) and 71% of patients were underexpansion [3]. The CRUISE (Can Routine Ultrasound Influence Stent Expansion) study showed that target vessel revascularization had been reduced by 44% and the final minimum stent area had been increased by 14% after the postdilation with the guidance of intravascular ultrasound [32]. During the bioresorbable vascular scaffolds and sirolimus-eluting stents implantation, the postdilation with high-pressure noncompliant balloon and large size balloon (balloons > 1 mm larger than the stent nominal size) also demonstrated safe clinical and angiographic results [33, 34]. There were a few studies on the postdilation at present, the majority of them were observational studies and excluded patients with ACS; only patients with stable coronary artery disease, long lesions, or calcification lesions were included. Recently, a meta-analysis (conference abstract) including seven observational studies for patients with coronary artery disease indicated that the postdilation could not reduce the risk of MACE, all-cause death, myocardial infarction, and target vessel revascularization and recommended that the postdilation should be performed in selective patients but not in all patients undergoing PCI [10]. However, due to the high proinflammatory risk, thrombotic environment, and coronary spasm caused by circulating vasoconstrictors in patients with acute myocardial infarction, the conclusions of postdilation in patients with coronary artery disease can not be extended to patients with ACS. Therefore, it is necessary to explore the benefits of postdilation in patients with ACS.

This meta-analysis suggested a trend to reduce MACE, which was similar to the results of previous meta-analysis [10, 35]. MACE was defined as cardiac death, target lesion-related myocardial infarction, or ischemia-driven target lesion revascularization in the meta-analysis by Hong et al. [35]. Although they concluded that the effect of postdilation on MACE was not statistically significant, the results suggested that postdilation tended to reduce the risk of MACE. Similarly, Chen et al. found that there seemed to be a downward trend on MACE after postdilation, but detailed definition of MACE was not provided in that meta-analysis [10]. Moderate heterogeneity of MACE was demonstrated in our study. The sensitivity analysis of MACE showed that the heterogeneity was derived from the study by Karjalainen et al. [14]. The conclusion of MACE changed statistically after exclusion of the study, and postdilation was considered to reduce MACE. Karjalainen et al. used single-blinded, randomized trial design and followed for 5 years, but other studies included in this outcome were observational studies and followed for 6 months to 2 years. Different trial design method and follow-up duration may be the reason for obvious heterogeneity in the study by Karjalainen et al. Therefore, more randomized trials and longer follow-up duration are needed to confirm the clinical impact of postdilation. The results of target vessel revascularization and myocardial infarction in our meta-analysis were also similar to that in the study by Chen and Li [10], while showing obvious heterogeneity in our study. The sensitivity analysis of target vessel revascularization found that the heterogeneity came from the study of Gao et al. [23]. The target lesions were more complex and immediate TIMI flow was impaired in the postdilation group in this study; finial TIMI flow was the same in two groups due to the use of intracoronary vasodilator agents. However, it may be associated with further adverse clinical outcomes. Meanwhile, the subgroup analysis showed that the target vessel revascularization could be reduced after the postdilation within 12 months, and patients with any ACS could also benefit from this strategy. The strategy of postdilation tended to increase the risk of myocardial infarction in this meta-analysis, although whether there is a postdilation or had no statistical difference on myocardial infarction. Therefore, more studies are needed to expand sample size to confirm this result. The sensitivity analysis of myocardial infarction displayed that the heterogeneity was derived from the study by Imori et al. [12]. The bioabsorbable scaffolds were used in this study, which was different from drug-eluting stents and bare-metal stents used in other studies. The postdilation increased myocardial infarction after excluding this study, which indicated that the postdilation was more suitable for bioabsorbable scaffolds. This may be due to the fact that the stent platform materials of bare-metal stent and drug-eluting stent are stainless steel, chrome-cobalt, platinum–chromium, or nickel/titanium alloy, which have a stable structure that provides reliable, compliant struts expansion without the risk of disruption. However, the bioabsorbable scaffolds use polylactic acid and other polymer materials as scaffolds to provide temporary mechanical support for stenotic or occluded coronary arteries. It represents a potential risk for clinical outcomes because of the relatively thick struts and limited expansion. Five-year follow-up from the ABSORB III Trial indicated that rate of target lesion failure was increased compared with everolimus-eluting stents [36]. The postdilation after the use of bioabsorbable scaffolds appears to be effective. Meanwhile, this meta-analysis suggested that the postdilation did not reduce thrombosis, which was consistent with the conclusion of Hong et al. in 2017 (HR = 0.39, CI 0.07–2.31, *P* = 0.279) [35]. The study by Chen et al. also found that the postdilation did not change all-cause death, which was different from the conclusion that the postdilation increased all-cause death in this study. This may be related to the inclusion criteria of that study [10]. All patients with coronary artery disease undergoing PCI were included in that meta-analysis, including patients with stable coronary artery disease. The levels of troponin I and highly sensitive C-reactive protein were elevated after stent expansion, suggesting more myocardial damage and inflammation [37]. The primary lesion in patients with acute coronary syndrome is more unstable due to more necrotic cores and fewer fibrous fatty plaques than in patients with stable coronary artery disease [38]; the elevation of cardiac troponin occurs in lesions with a large necrotic core area and in lipid-rich lesions [39, 40]. Therefore, the deployment of postdilation in patients with ACS will increase the risk of myocardial damage, which may be the reason for increased all-cause death in this meta-analysis. In addition, studies have shown that the levels of plasma B-type natriuretic peptide significantly increase following the postdilation, which is a biomarker of heart failure [37]. Long-term heart failure can also cause an increased death. Besides, different antiplatelet therapy regimens also affect clinical outcomes. However, precise mechanism remains unclear. Interestingly, there was no increase in all-cause death in ACS patients within 12 months after postdilation. We considered this may be related to the antiplatelet regimen. Dual antiplatelet therapy is mainly used for ACS patients after PCI for 12 months, while aspirin monotherapy or dual antiplatelet therapy regimen should be depended on the specific conditions of patients 12 months later, which may lead to late ischemia or bleeding events and affect long-term all-cause death. Moreover, previous studies lacked a uniform definition of reflow, making no reflow rate and slow reflow rate fluctuate between 1% and 30% [24], which made it difficult to guide clinical practice. Therefore, this study unified the definition of no reflow and slow reflow and concluded that the postdilation had no effect on no reflow and slow reflow.

Angiography is suboptimal for identifying stent underexpansion and malapposition; the use of intravascular imaging devices can overcome these limitations. A previous study has showed that the postdilation can increase the minimum in-stent area and reduce revascularization under guidance of intravascular ultrasound (IVUS) [9]; a subsequent study also found that the postdilation was used more frequently during IVUS-guided stent implantation to improve stent underexpansion compared with angiography [41]. However, IVUS-guided stent implantation is not widespread currently, and optical coherence tomography (OCT) guided stent implantation is rarely studied. Angiography is still the most commonly used imaging method to guide surgical strategy. In our meta-analysis, a few patients underwent intravascular imaging. Only 20% of IVUS and 3% of OCT were used in the study by Imori et al. [12], and the rest of patients still received angiography to guide stent implantation. In addition, some studies have shown that IVUS and OCT have comparable cost-effectiveness, and both of them are higher than angiography [42, 43]. Economic factors may also influence the use of intravascular imaging devices. At present, some studies evaluated the clinical and angiographic results of postdilation under angiography and reached different conclusions. We considered that, under the guidance of angiography, balloon dilation after stent implantation has certain clinical value.

However, these results should be interpreted carefully. Firstly, whether or not to receive the postdilation strategy after stent deployment mainly depends on the individual situation of patients and the wills of operator. Secondly, the complications of PCI are not only related to the stent underexpansion, but also to thrombus aspiration, thrombolytic drugs for intracoronary injection or vasodilator, and antiplatelet drug compliance. Finally, although some studies have shown coronary TIMI flow grade after PCI, only a few studies compared no reflow or slow reflow between two groups. Therefore, further randomized controlled trials are needed to verify the benefits of postdilation.

## 5. Limitations

The limitations of this study should be recognized. Firstly, this meta-analysis mainly included retrospective studies (7/11), which were more likely to include selection, observation, or publication bias, and confounding factors. Due to the limitation of the number of postdilation studies, studies with different designs and sample sizes should not be excluded. Secondly, the definition of outcome varies in each study, and the definition from the original study was adopted. Thirdly, three post hoc analyses were included in this study, which might have lost some of the raw data. Fourthly, the benefit of postdilation may vary among different types of stent; as detailed data on bare-metal stents, drug-eluting stents, and bioresorbable scaffolds of study included were not available in this meta-analysis, a subgroup analysis of stent type was not performed. Finally, patients are expected to receive dual antiplatelet therapy for at least one year after PCI in the studies included, but the details of whether patients regularly take antiplatelet agents are still unclear, which is also the key to clinical efficacy in the future.

## 6. Conclusion

In conclusion, the postdilation strategy tends to reduce MACE in patients with ACS; despite significantly increasing all-cause death, no significant difference exists in other clinical outcomes and coronary TIMI flow grade. However, more specialized randomized controlled trials are demanded for confirming this conclusion.

## Figures and Tables

**Table 1:** Baseline characteristics of studies included.

Study	Follow-up duration	N (n)	STEMI (n)	Mean age (y)	Male (%)	Hypertension (%)	Hyperlipidemia (%)	Diabetes (%)	Current smoking (%)	DES (%)	Stent length (mm)	Stent diameter (mm)
Aadat et al. 2019	348 ds	500/724	500/724	58.4 ± 11.4/57.0 ± 12.1	76.0/77.0	60/54	60.0/60.0	28.0/26.0	8.0/11.0	74.5/70.9	26.5 ± 8.5/22.5 ± 6.7	3.0 ± 0.3/3.1 ± 0.4
Gao et al. 2018	12 M	199/137	199/137	59.4 ± 10.9/61.0 ± 11.3	78.9/76.6	61.3/52.6	3.0/2.9	NA	62.3/66.4	100/100	30.84 ± 12.17/27.86 ± 10.11	NA
Karjalainen et al. 2017	5 Y	357/470	135/186	63.6 ± 11.3/62.5 ± 12.3	75.9/76.2	50.7/49.6	45.4/58.9	82.1/83.8	67.5/65.5	100/100	18.8 ± 5.3/17.8 ± 5.5	3.18 ± 0.44/3.12 ± 0.44
Tasal et al. 2013	6 M	214/191	214/191	57.5 ± 11.8/56.4 ± 13.0	73.4/75.9	30.8/30.4	49.5/52.9	18.7/22.5	41.2/35.5	100/100	23.4 ± 6.2/20.5 ± 5.9	2.9 ± 0.4/3.1 ± 0.4
Biswas et al. 2012	10.5 M	71/89	71/89	61.0 ± 12.6/62.9 ± 14.1	78.9/75.3	43.7/60.7	35.2/48.3	22.5/25.8	46.5/40.5	26.8/23.6	NA	NA
Zhang et al. 2010	12 M	586/772	316/413	61.7 ± 12.9/60.1 ± 12.7	62.8/63.0	67.2/63.9	NA	2.60/23.0	NA	57.9/48.6	NA	NA
Imori et al. 2016	2 Y	148/153	63/57	60.1 ± 12.7/61.0 ± 12.8	80.4/74.5	57.4/63.2	50.7/31.6	10.1/13.8	45.9/50.0	0	22.2 ± 5.4/18.3 ± 3.8	3.1 ± 0.4/3.1 ± 0.4
Wang et al. 2019	1 Y	228/114	228/114	61.7 ± 8.4/63.6 ± 6.8	68..9/76.3	41.2/46.5	18.4/14.9	26.8/22.8	43.9/69.3	NA	29.6 ± 7.1/23.7 ± 5.9	3.2 ± 1.7/3.1 ± 1.3
Qin et al. 2019	1 M	76/319	76/319	61.7 ± 12.3/60.0 ± 12.3	82.9/82.5	54.0/51.4	38.2/36.7	23.7/18.5	44.7/18.5	NA	NA	NA
Soylu et al. 2018	No	62/62	62/62	60.9 ± 13.2/60.2 ± 13.9	75.8/72.6	56.5/50.0	NA	30.6/27.4	59.7/58.1	100/100	26.75 ± 7.65/24.68 ± 7.35	3.04 ± 0.46/2.99 ± 0.44
Yamaji et al. 2017	No	73/118	73/118	59.4 ± 9.7/58.2 ± 10.4	80.8/83.5	39.7/39.8	NA	16.4/16.9	54.8/44.9	34.2/60.2	NA	NA

STEMI: ST-elevation myocardial infarction; DES: drug-eluting stent; D: day; M: month; Y: year; NA: not available.

**Table 2:** Procedural characteristics of studies included.

	Saadat et al. 2019	Qin et al. 2019	Gao et al. 2018	Soylu et al. 2018	Yamaji et al. 2017∗	Tasal et al. 2013	Biswas et al. 2012
N	500/724	76/319	199/137	62/62	73/118	214/191	71/89
Culprit lesion-related artery (%)							
LM	NA	4.0/1.3	NA	NA	NA	NA	NA
LAD	63.8/44.6	40.8/51.1	48.7/41.6	43.5/48.4	31.5/43.3	43.9/54.5	NA
LCX	10.4/15.2	19.7/8.5	13.1/7.3	11.3/8.1	13.7/16.7	25.7/20.4	NA
RCA	23.2/35.5	35.5/39.2	38.2/51.1	45.2/43.5	54.8/40.0	30.4/25.1	NA
Pre-PCI TIMI flow							
0/1	309/518	63/285	155/103	43/45	48/81	160/148	44/58
2	106/135	13/34	15/17	10/10	8/13	45/23	16/17
3	85/71	0/0	29/17	9/7	19/25	9/10	11/14
Post-PCI TIMI flow							
0/1	1/5	1/3	4/1	2/0	0/0	5/4	0/0
2	159/210	2/15	23/25	20/9	0/2	49/34	11/15
3	340/509	73/300	172/111	40/53	73/118	160/153	60/74

LM: left main; LAD: left anterior descending; LCX: left circumflex; RCA: right coronary artery; TIMI: thrombolysis in myocardial infarction; PCI: percutaneous coronary intervention. ∗Number of lesions, *n* = 120.

## Data Availability

All data used to support the findings of our study are included within the article.

## Supplementary Materials

Supplementary Materials Table S1: the definition of MACE in studies included. Table S2: quality assessment scale of Newcastle-Ottawa Scale (NOS) for nonrandomized studies. Figure S1: risk of bias of the studies included by Cochrane risk assessment tool. Figure S2: sensitivity analysis of MACE, myocardial infarction, and target vessel revascularization between postdilation and non-postdilation groups. Figure S3: sensitivity analysis of myocardial infarction and target vessel revascularization between postdilation and non-postdilation groups after removing the studies of Karjalainen et al., Imori et al., and Gao et al. Figure S4: subgroup analyses of MACE, all-cause death, stent thrombosis, myocardial infarction, and target vessel revascularization according to classification of diseases (STEMI or any ACS) and duration of follow-up (<12 months or ≥12 months).
