# Supplementary material for: The Clinical and Angiographic Outcomes of Postdilation after Percutaneous Coronary Intervention in Patients with Acute Coronary Syndrome: A Systematic Review and Meta-Analysis
Source: J Interv Cardiol. 2021 Apr 9;2021:6699812. doi: 10.1155/2021/6699812 (PMC8055432; doi:10.1155/2021/6699812)
Supplement: Supplementary Materials — Table S1: the definition of MACE in studies included. Table S2: quality assessment scale of Newcastle-Ottawa Scale (NOS) for nonrandomized studies. Figure S1: risk of bias of the studies included by Cochrane risk assessment tool. Figure S2: sensitivity analysis of MACE, myocardial infarction, and target vessel revascularization between postdilation and non-postdilation groups. Figure S3: sensitivity analysis of myocardial infarction and target vessel revascularization between postdilation and non-postdilation groups after removing the studies of Karjalainen et al., Imori et al., and Gao et al. Figure S4: subgroup analyses of MACE, all-cause death, stent thrombosis, myocardial infarction, and target vessel revascularization according to classification of diseases (STEMI or any ACS) and duration of follow-up (<12 months or ≥12 months). [file 6699812.f1.docx]

**The clinical and angiographic outcomes of post-dilation after percutaneous coronary intervention in patients with acute coronary syndrome: a systematic review and meta-analysis**

Yan Li^1#^, MD; Xiying Liang^1#^, MD; Wenjiao Zhang^1#^, MD; Xuan Qiao^1#^, MD; Zhilu Wang^2*^, MD

^1^ The First Clinical Medical College of Lanzhou University, Lanzhou, Gansu, China;

^2^ Department of Cardiology, The First Hospital of Lanzhou University, Lanzhou, Gansu, China.

#These authors contributed equally to this work.

*Corresponding author: Zhilu Wang, Department of Cardiology, The First Hospital of Lanzhou University, Lanzhou, Gansu, China. E-mail: [wangzhl@lzu.edu.cn](mailto:wangzhl@lzu.edu.cn)

**Supplementary Table S1.** The definition of MACE in studies included.

| Study | The definition of MACE |
| --- | --- |
| Saadat et al. 2019 | cardiac death + nonfatal myocardial infarction + repeat revascularization |
| Wang et al. 2019 | cardiac death + myocardial infarction+ revascularization + stent thrombosis |
| Karjalainen et  al. 2017 | cardiac death + nonfatal myocardial infarction + target lesion revascularization， |
|  |  |
| Imori et al. 2016 | all-cause death + myocardial infarction + target lesion revascularization |
| Tasal et al. 2013 | death + target vessel revascularization + stent thrombosis |
| Biswas et al. 2012 | cardiac death + myocardial infarction + target vessel revascularization |

**Supplementary Figure S1.** Risk of bias of the studies included by Cochrane risk assessment tool.

**Supplementary Table S2.** Quality assessment scale of Newcastle-Ottawa Scale (NOS) for non-randomized studies.

| Study | Saadat et al. 2019 | Wang et al. 2019 | Qin et al. 2019 | Gao et al. 2018 | Imori et al. 2016 | Tasal et al. 2013 | Biswas et al. 2012 | Zhang et al. 2010 |
| --- | --- | --- | --- | --- | --- | --- | --- | --- |
| Representativeness of the exposed cohort (1) | A | A | A | A | A | A | A | A |
| Selection of the non-exposed  cohort (1) | A | A | A | A | A | A | A | A |
| Ascertainment of exposure (1) | A | A | A | A | A | A | A | A |
| Outcome of interest not present at start (1) | B | B | B | B | B | B | B | B |
| Comparability (2) | A | A | A | A | A | A | A | A |
| Assessment of outcome (1) | B | B | B | B | B | B | B | B |
| Follow-up duration (1) | A | A | B | A | A | B | B | A |
| Adequacy follow up (1) | A | A | A | A | A | A | A | A |

Notes: (1) Representative-ness of the exposed cohort: A, truly representative of the average patient with post-dilation after stent implantion; B, somewhat representative of the average patient with post-dilation after stent implantation; C, selected special group; D, no description of the derivation of the hort. (2) Selection of the non-exposed cohort: A, drawn from the same community as the exposed cohort; B, drawn from a different source; C, no description of the derivation of the non-exposed cohort. (3) Ascertainment of exposure: A, secure record (e.g., surgical records); B, structured interview; C, written self-report; D, no description. (4) Demonstration that outcome of interest was not present at start of study: A, yes; B, no. (5) Comparability of cohorts on the basis of the design or analysis: A, study controls for comorbidities. B, study controls for additional risk factors; C, not done. (1) Assessment of outcome: A, independent blind assessment; B, record linkage; C, self-report; and D, no description. (2) Was follow-up long enough for outcomes to occur: A, yes; B, no. (3) Adequacy of follow-up of cohort: A, complete follow-up all subjects accounted for; B, subjects lost to follow-up unlikely to introduce bias (small number lost), follow-up rate higher than 90%, or description provided of those lost; C, follow-up rate 90% or lower(select an adequate percentage) and no description of those lost; and D, no statement.

**a. MACE**

**
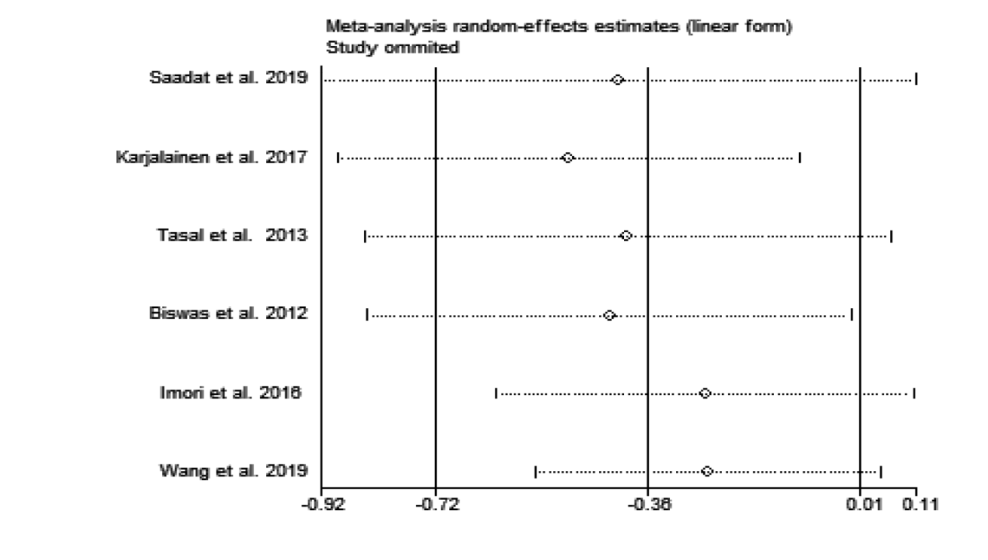
**

**b. Myocardial infarction**

**
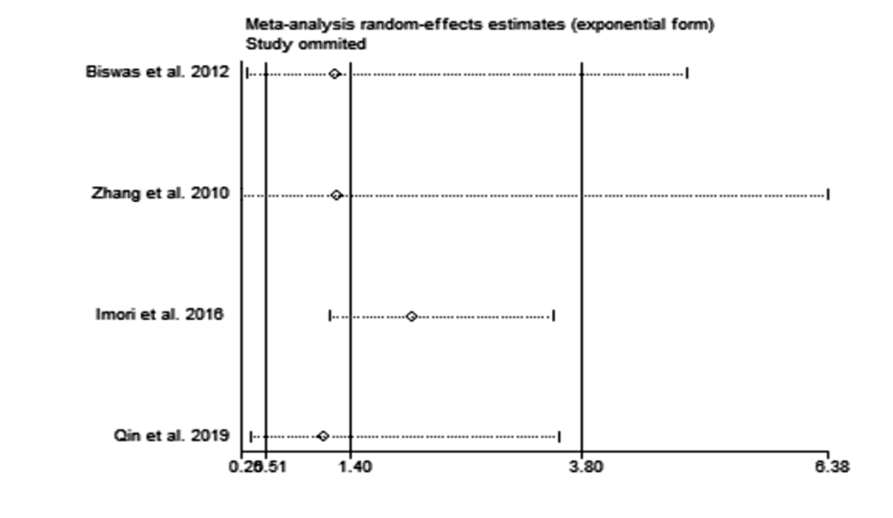
**

**c. Target vessel revascularization .**

**
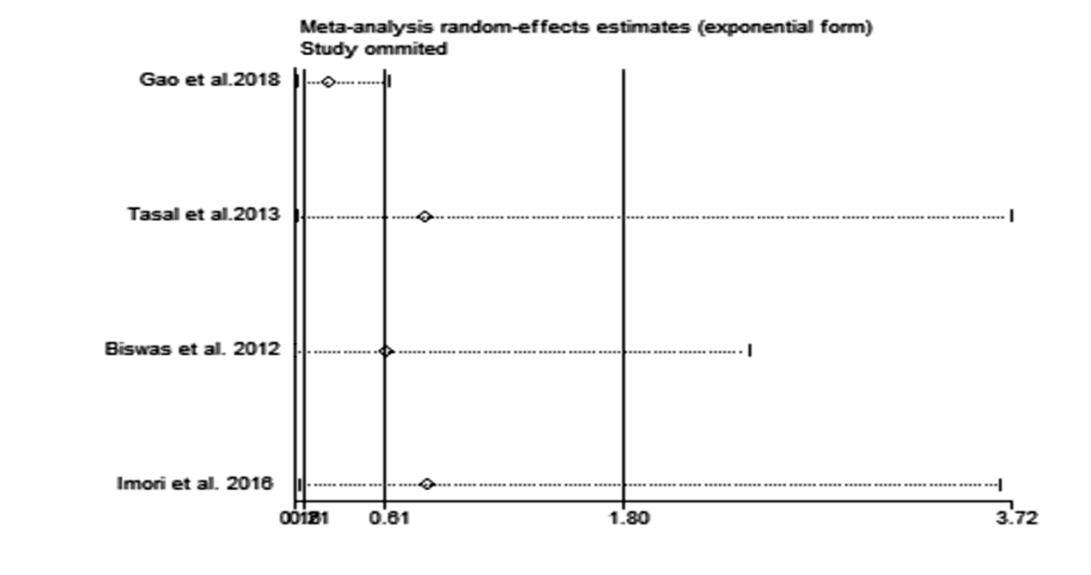
**

**Supplementary Figure S2.** Sensitivity analysis of MACE, myocardial infarction, and target vessel revascularization between post-dilation and non-post-dilation groups.

**a. MACE**

**b. Myocardial Infarction**

**c. Target Vessel Revascularization**

**Supplementary Figure S3.** Sensitivity analysis of myocardial infarction and target vessel revascularization between post-dilation and non-post-dilation groups after removing the studies of Karjalainen et al, Imori et al, and Gao et al.

**a. MACE**

**b. All-cause death**

**Stent thrombosis**

**Myocardial infarction**

**Target vessel revascularization**

**c. All-cause death**

**Stent thrombosis**

**Myocardial infarction**

**Target vessel revascularization**

**Supplementary** **Figure S4.** Subgroup analyses of MACE, all-cause death, stent thrombsis, myocardial infarction, and target vessel revascularization according to classification of diseases (STEMI or any ACS) and duration of follow-up (<12 months or≥12 months).

**Search strategy**  (Take the PubMed database for an example)

#1 "Acute Coronary Syndrome"[MeSH Terms] OR "Acute Coronary Syndromes"[Title/Abstract] OR "Acute Coronary Syndrome"[Title/Abstract] OR "coronary syndrome acute"[Title/Abstract] OR "coronary syndromes acute"[Title/Abstract] OR "syndrome acute coronary"[Title/Abstract] OR "syndromes acute coronary"[Title/Abstract] OR "ACS"[Title/Abstract]

#2 "Myocardial Infarction"[MeSH Terms] OR "Myocardial Infarction"[Title/Abstract] OR "Myocardial Infarctions"[Title/Abstract] OR "infarction myocardial"[Title/Abstract] OR "infarctions myocardial"[Title/Abstract] OR "Cardiovascular Stroke"[Title/Abstract] OR "stroke cardiovascular"[Title/Abstract] OR "strokes cardiovascular"[Title/Abstract] OR "Heart Attack"[Title/Abstract] OR "Heart Attacks"[Title/Abstract] OR "Myocardial Infarct"[Title/Abstract] OR "Myocardial Infarcts"[Title/Abstract] OR "infarct myocardial"[Title/Abstract] OR "infarcts myocardial"[Title/Abstract] OR "heart infarction"[Title/Abstract] OR "MI"[Title/Abstract] OR "acute myocardial infarction"[Title/Abstract] OR "AMI"[Title/Abstract]

#3 "Percutaneous Coronary Intervention"[MeSH Terms] OR "Percutaneous Coronary Intervention"[Title/Abstract] OR "Percutaneous Coronary Interventions"[Title/Abstract] OR "coronary intervention percutaneous"[Title/Abstract] OR "coronary interventions percutaneous"[Title/Abstract] OR "intervention percutaneous coronary"[Title/Abstract] OR "interventions percutaneous coronary"[Title/Abstract] OR "Percutaneous Coronary Revascularization"[Title/Abstract] OR "Percutaneous Coronary Revascularizations"[Title/Abstract] OR "coronary revascularization percutaneous"[Title/Abstract] OR "revascularization percutaneous coronary"[Title/Abstract] OR "revascularizations percutaneous coronary"[Title/Abstract] OR "PCI"[Title/Abstract]

#4 "Stents"[MeSH Terms] OR "stent"[Title/Abstract] OR "Stents"[Title/Abstract] OR "stenting"[Title/Abstract]

#5 "Angioplasty"[MeSH Terms] OR "Angioplasty"[Title/Abstract] OR "Angioplasties"[Title/Abstract] OR "Endoluminal Repair"[Title/Abstract] OR "Endoluminal Repairs"[Title/Abstract] OR "repair endoluminal"[Title/Abstract] OR "Percutaneous Transluminal Angioplasty"[Title/Abstract] OR "angioplasty percutaneous transluminal"[Title/Abstract] OR "transluminal angioplasty percutaneous"[Title/Abstract] OR "angioplasty transluminal"[Title/Abstract] OR "Transluminal Angioplasty"[Title/Abstract]

#6 "postdilation"[Title/Abstract] OR "post-dilation"[Title/Abstract] OR "Postdilatation"[Title/Abstract] OR "Post-dilatation"[Title/Abstract]

#7 (#1 OR #2) AND (#3 OR #4 OR #5) AND #6
